# Cost-Effective Control of Plant Disease When Epidemiological Knowledge Is Incomplete: Modelling Bahia Bark Scaling of Citrus

**DOI:** 10.1371/journal.pcbi.1003753

**Published:** 2014-08-07

**Authors:** Nik J. Cunniffe, Francisco F. Laranjeira, Franco M. Neri, R. Erik DeSimone, Christopher A. Gilligan

**Affiliations:** 1 Department of Plant Sciences, University of Cambridge, Cambridge, United Kingdom; 2 Embrapa Cassava and Fruits, Cruz das Almas, Brazil; Pennsylvania State University, United States of America

## Abstract

A spatially-explicit, stochastic model is developed for Bahia bark scaling, a threat to citrus production in north-eastern Brazil, and is used to assess epidemiological principles underlying the cost-effectiveness of disease control strategies. The model is fitted via Markov chain Monte Carlo with data augmentation to snapshots of disease spread derived from a previously-reported multi-year experiment. Goodness-of-fit tests strongly supported the fit of the model, even though the detailed etiology of the disease is unknown and was not explicitly included in the model. Key epidemiological parameters including the infection rate, incubation period and scale of dispersal are estimated from the spread data. This allows us to scale-up the experimental results to predict the effect of the level of initial inoculum on disease progression in a typically-sized citrus grove. The efficacies of two cultural control measures are assessed: altering the spacing of host plants, and roguing symptomatic trees. Reducing planting density can slow disease spread significantly if the distance between hosts is sufficiently large. However, low density groves have fewer plants per hectare. The optimum density of productive plants is therefore recovered at an intermediate host spacing. Roguing, even when detection of symptomatic plants is imperfect, can lead to very effective control. However, scouting for disease symptoms incurs a cost. We use the model to balance the cost of scouting against the number of plants lost to disease, and show how to determine a roguing schedule that optimises profit. The trade-offs underlying the two optima we identify—the optimal host spacing and the optimal roguing schedule—are applicable to many pathosystems. Our work demonstrates how a carefully parameterised mathematical model can be used to find these optima. It also illustrates how mathematical models can be used in even this most challenging of situations in which the underlying epidemiology is ill-understood.

## Author Summary

We consider how mathematical models can be used to inform the control of plant disease, even when the identity and biology of the pathogen are not well understood. This is often the case: control of emerging epidemics is most likely to have a significant effect when epidemics remain small, but little may then be known. We analyse data from an experimental plot concerning spread of Bahia bark scaling of citrus, an economically-important disease in north-eastern Brazil, by fitting a mathematical model, which also accounts for uncertainty, to disease spread. Our model captures the epidemiological features of the disease, revealing that transmission is localised and that disease spreads relatively slowly. We use the model to investigate fundamental trade-offs underlying cultural disease control at scales relevant to citrus production. We show how optimal planting densities can be defined, which balance slower spread of disease against the profit that would be lost by growing fewer plants. We also show how the cost of looking for and removing symptomatically diseased plants can be balanced against the reduced disease it leads to. Ours is the first study to consider how a parameterised mathematical model can be used to design optimised cultural controls of plant disease.

## Introduction

Mathematical models of plant disease can be used to screen and assess control strategies [1]–[10]. Although work on plants is not subject to the ethical concerns that hamper experimentation targeting pathogens of animal or human hosts, mathematical modelling nevertheless becomes particularly compelling for plant diseases when logistic constraints mean that experimentation would be costly or difficult. This situation is exemplified by diseases caused by pathogens with epidemiology necessitating long experiments to yield useful data [11]–[13], pathogens causing symptoms that are difficult to detect [14], [15], pathogens with epidemiology that is ill-understood [16], [17], and/or pathogens that would require experimental trials in the vicinity of susceptible commercial growing operations [18], [19].

Here we develop a model of Bahia bark scaling of citrus (BBSC) on grapefruit, a pathosystem subject to each of these logistical constraints. BSSC has been endemic to north-eastern Brazil since the 1960s [20], but its etiology remains unknown [21]. We use Markov chain Monte Carlo with data augmentation [22] to fit a spatially-explicit, stochastic, epidemiological model to a data-set charting the spread of BBSC through a small experimental grove. We go on to alter the host topology and parameters in this model to use it to assess the efficiency and cost-effectiveness of control at the scale of a typical grove as used in citrus production in Brazil. As little is known of the putative BBSC pathogen, and even less about any potential vector, it is difficult to reliably estimate the efficacy of any chemical [23] or biological [24] control. We therefore concentrate on cultural strategies [25], and focus on the effectiveness of reducing the density of planting [26] and of roguing [10] (i.e. searching for and removing infected plants).

The spread of plant pathogens is typically localised, and so it is intuitive that the progression of disease through a host population will be affected by planting density. Direct as well as indirect effects of host density on disease incidence have been proposed [26], and lower host densities are almost always associated with lower levels of disease [27]. Indeed the “dilution effect” caused by increased distances between pairs of hosts has been suggested to underlie the success of crop mixtures [28] and intercropping [29], although other more complex mechanisms are thought to be involved in both cases [30]–[33]. However, there are very few models specfically targeting the effect of host density on disease spread. Despite work concentrating on how percolation thresholds can be related to the distance between pairs of nearest neighbours [34], [35], tests of that theory have largely been restricted to small-scale model systems [36], and application to real pathosystems remains in its infancy [37], [38]. Percolation is also only strictly relevant to systems where spread is restricted to nearest neighbour transmission, although this does map well to the soil-borne pathogens that are the focus of that work. Other work has concentrated on how host density affects invasion thresholds [39], [40], but does not provide a clear prescription for how to optimise host densities when disease is able to invade. While there have also been studies showing how the landscape-scale dynamics of disease are conditioned on the configuration and availability of patches of suitable habitat [41], or fields planted with susceptible varieties [42], that work offers little at scales relevant to individual farmers or growers.

Roguing is commonly used for systemic diseases of high-value or perennial crops [43], particularly when labour is cheap compared with the cost of chemicals [44], or for pathogens which cannot be effectively controlled by chemical means [18], [19]. Viral pathogens for which roguing is practised include cassava mosaic [45], bunchy top of banana [46], cocao swollen shoot [47], [48], citrus tristeza [49], plum pox [50] and sweet potato chlorotic stunt [51], although roguing is also used for bacterial pathogens (e.g. almond leaf scorch, caused by *Xylella fastidiosa* [52]), and for fungal diseases (e.g. lettuce drop, caused by *Sclerotinia minor* [53]). The only constraint is that pathogens must cause symptoms that can be detected, either by visual inspection or by diagnostic testing. Roguing has been included in non-spatial mathematical models for a number of years [1], [2], [54], [55], and more recent work has embedded control by roguing in spatial models of pathogen spread [10], [56]–[58], although realistic parameterisation of pathogen dispersal is less common [6]–[8]. Typically these later models have also considered culling, in which all hosts within a particular distance of a symptomatic focal host are removed at the time of control. Some of these models [57], [58] have explicitly included economics, although the focus has been the cost of treatment (i.e. the cost of removal of diseased host plants). For perennial hosts that are not replanted, however, the cost of detection may, in fact, be more important, since an individual host can be removed at most once, but may be examined for symptoms any number of times. The only model to include detection costs used optimal control theory to show rigorously how to balance the costs of detection and control within a fixed budget [59], but the mathematical complexity of this procedure necessarily restricted attention to a non-spatial, deterministic model. There are no examples of a model-based approach that optimises the economic aspects of roguing including the cost of detection via a model parameterised to spread data.

We have taken advantage of the availability of experimental data for model fitting to frame our analyses specifically in terms of the dynamics and control of BBSC. However, the controls we examine are widely used, and the techniques we use in our modelling and fitting are applicable to a large number of pathosystems. We therefore prefer to think of the BBSC system as a data-driven case study that provides an opportunity to address the following more general questions.

1. Can we use a model to describe the spread of a disease even when its detailed etiology is unknown?
2. Can we parameterise the model using experimental results to allow us to scale-up and make predictions at agriculturally-relevant scales?
3. How can the financial benefit of effective cultural control be balanced against its inherent cost?

## Materials and Methods

### Bahia bark scaling of citrus

BBSC affects most citrus species and varieties, but is especially severe on grapefruits [60]. Symptoms appear similar to Citrus Psorosis A, and include darkening and thickening of the bark leading to scaling lesions on the trunk and branches, dieback of young branches, and significant gum extrusion. However leaf symptoms on inoculated indicator plants, together with histopathological and molecular studies, indicate BBSC is a distinct disease. The study of Laranjeira *et al.* [20] resulted in the only published data focusing on BBSC spread (see Text S1). It demonstrated that the disease is polyetic and naturally transmitted. The speed of disease spread and the pattern of dispersal appear consistent with an insect vector of limited dispersion ability. However the identity of this putative vector is unclear, as is the identity of the pathogen itself [21].

BBSC currently remains restricted to two states in the Brazilian north east, Bahia and Sergipe [21], [61]. Since dispersal is thought to be localised, the principal risk of an epidemic arising elsewhere in Brazil is likely to occur by transplanting infectious plants. Introduction of BBSC by inadvertent transplantation is certainly possible: Santos *et al.* [62] have described BBSC symptoms in plants used for budwood in Bahia, Brazil. There is therefore a need to understand whether and how a spatially-isolated epidemic could be effectively controlled. This must be done even though our biological understanding of the epidemiology of BBSC remains limited.

### Epidemiological model

**Figure 1:**
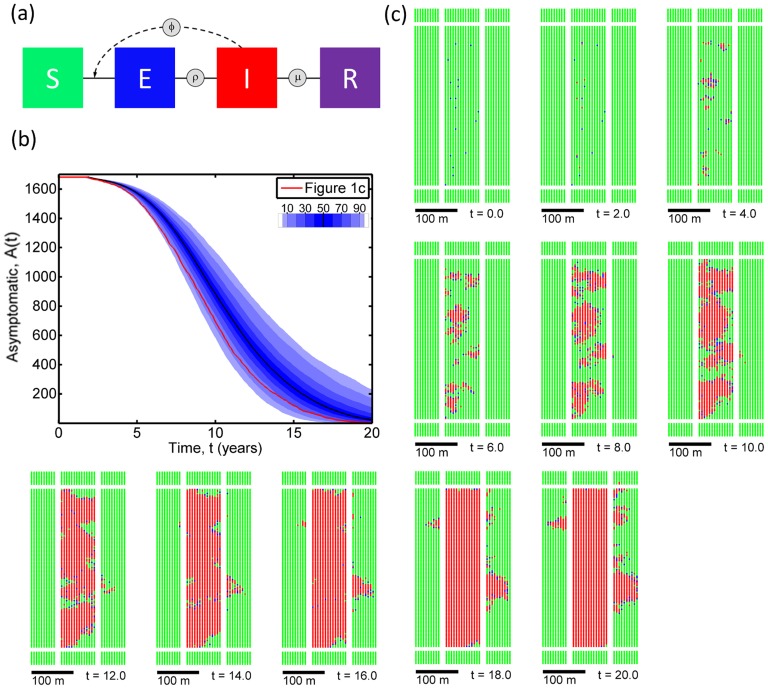
The underlying model and typical results without control. (a) The compartmental structure of the (S)usceptible, (E)xposed, (I)nfected, (R)emoved model. (b) Spread of disease in a typical grove when there is no control, showing the number of asymptomatic plants within the central grove (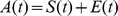) as a function of time, 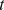, starting with 1% of hosts (i.e. 17 plants) exposed to the pathogen at 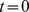, and sampling parameters 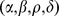 randomly on each run independently from the joint posterior parameter distribution obtained in model fitting. The density of shading shows the distribution of 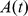 at each value of 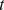 (1000 independent simulations). Breaks between different colours are at the 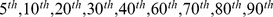 and 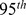 percentiles, with the 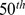 percentile marked by the black curve. (c) Snapshots of disease spread from the single realisation shown by the red curve in Figure 1(b); green corresponds to healthy trees (S), blue to trees that have been infected but are not yet infectious (E), and red to trees that are able to infect other trees (I). Since there is no control, no trees enter the (R)emoved compartment.

We use a spatially-explicit, stochastic, compartmental SEIR model [4] to represent BBSC dynamics at the scale of a grapefruit grove. Individual host plants are categorised by disease status: (S)usceptible hosts are uninfected; (E)xposed hosts are latently infected, and so are neither symptomatic nor infectious; (I)nfected hosts are both infectious and symptomatic; and (R)emoved hosts have been removed by control (Figure 1(a)). The E to I transition occurs at rate 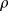, corresponding to average latent period 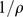 (see also Table S1). Since infectious hosts are always symptomatic in our model, the average incubation period is also 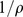. Infected hosts do not appear to suffer increased mortality due to BBSC infection [20], and so in the absence of control the rate of transition from the I to R compartment is fixed at zero. However, if control by roguing is included in the model, the removal rate is set by how frequently and efficiently infected plants are detected and removed, with rounds of detection and removal according to a schedule that is fixed in advance. Since we work over a twenty year timescale, similar to the typical productive lifespan of an individual citrus host [63], [64], we do not attempt to model natural death. We also do not consider replanting of any plants removed by roguing, since this is not common in the Brazilian citrus industry, perhaps due to growers' perception that replanting removed hosts would lead to a heterogeneous grove that would be more difficult to cultivate [63].

The rate of infection of susceptible hosts depends on the disease status of all other hosts in the system. In particular, if host 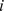 is susceptible at time 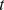, then it becomes latently infected (i.e. transitions to the E compartment) at rate 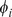, where
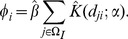
(1)


The summation runs over the set of all (I)nfectious hosts, 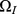, and 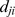 denotes the distance from infectious host 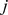 to susceptible host 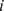. The parameter 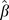 sets the rate of infection. Spatial dependency in spread is controlled by the dispersal kernel, 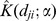. Here, noting the constant velocity of the epidemic front in the experimental grove [65], and following exploratory analyses that strongly supported the choice, we used the exponential kernel, normalized in two dimensions
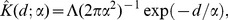
(2)where 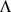 is the area of susceptible tissue presented by an individual host. The factor of 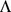 is included since, strictly-speaking, the underlying normalised dispersal kernel 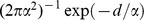 is a probability density function, with dimensions of inverse area, meaning the observed rate of infection must be calculated by integration over the area of the recipient plant. Assuming the kernel is constant over this area reduces the integration to a simple multiplication, and so leads to Equation (2) above [66], [67]. Since the infection rate 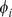 then depends entirely on the product 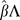 in Equation (1), we rescale the area of a single host into the infection rate, setting



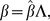
(3)

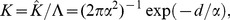
(4)

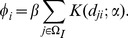
(5)


Our model fitting then estimates the value of 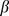 directly, since it is this product which sets the observed rate of spread of disease in our model. The mean distance of dispersal is 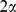 [68]. Since we model a grove that initially contains immature plants, and guided by the temporal pattern of disease spread in the experimental grove, we include a delay, 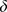, to allow young plants to reach epidemiological maturity [20], [21]. This delay prevents the disease from spreading for the first 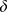 units of time, but otherwise does not affect the dynamics of infection in the model. Including this delay is therefore equivalent to considering two age classes of tree in the model: juveniles of age less than 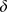, that cannot become infected or transmit infection, and adult trees of age greater than 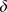, that are epidemiologically competent. The inclusion of this extra parameter was strongly supported by our model fitting (see Results and Text S2).

#### Host topology

The host landscape comprises a central grove and the adjoining ten rows/columns of the eight neighbouring groves (Figure 1(c)). The central grove contains 1680 trees, arranged in 14 rows of 120, at spacing 6 m
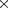
4 m. The distance between adjacent pairs of groves is 12 m. Spacings between individual trees and between pairs of groves reflect standard cultivation citrus patterns in Brazil [63]. We focus on the disease status of trees within the central grove, and although underlying pathogen dynamics are identical over the entire landscape, only the central grove is subject to any control. Since we include hosts in neighbouring groves, our model incorporates both secondary infection within the central grove, and infection due to the surrounding groves becoming infected and then re-exporting inoculum into the central grove (i.e. primary infection from the point of view of the target). This force of primary infection on the central grove varies over time, as the density of infection in neighbouring groves changes.

#### Initial infection

Initial infection is assumed to occur via transplantation into the central grove, planting one or more immature infected plants at 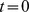 at random positions. These are set to be in the exposed compartment in the model and so are neither symptomatic nor infectious initially. We denote the percentage of exposed plants that are introduced at 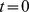 by 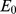, and we allow this quantity to vary, corresponding to a measure of how carefully new plantings are inspected for suspected symptoms of the disease.

### Parameter estimation from experimental data

**Figure 2:**
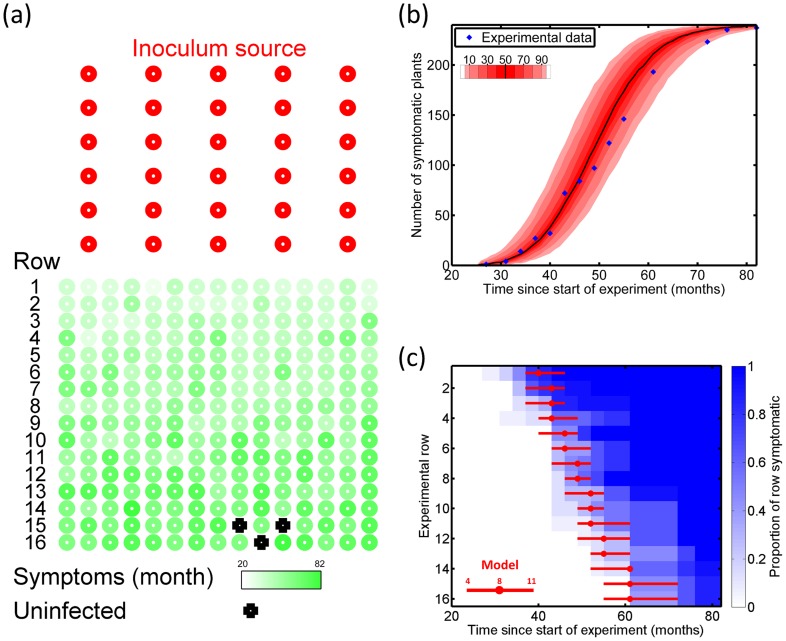
Goodness of fit. (a) The experimental grove. The red circles correspond to infected mature plants used as initial sources of inoculum, and the green circles correspond to the juvenile plants that were planted at 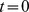 and that were available to become infected throughout the experiment (the density of shading shows the time at which symptoms emerged on each plant). The mature plants were at 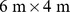 spacing; the immature plants at 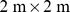 spacing; the closest distance between the two groups of plants was 5 m. (b) Comparing temporal spread of disease in the experiment with the results from an ensemble of simulation runs: the simulated distribution of the number of symptomatic plants at each time is shown by the density of red shading; the experimental data by blue dots. (c) Comparing spatial spread of disease in the experiment with the results from an ensemble of simulations: the density of blue shading shows the proportion of each row that was symptomatic by any particular time in the experiment; the red horizontal bars summarise the results of simulations. The median time at which the 4th and 11th plants in each row became symptomatic is shown by the end points of each red bar, and the red dot shows the median time at which the 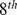 plant became symptomatic. All times from simulations were rounded up to the next date of sampling in the actual experiment to allow fair comparison with the discrete times of sampling used in the experimental protocol.

Data from the experiment of Laranjeira *et al.* [20] were used to fit the model. These data consist of successive snapshots over time, tracking the disease status of each host in a small experimental grove. This grove contained 240 grapefruit (*Citrus paradisi* Macf.) plants in 16 rows of 15. Immature plants were planted at regular 2 m
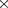
2 m spacing at the start of the experiment, at a closest distance of 5 m from twenty-five BBSC symptomatic adult grapefruit plants arranged in a rectangular lattice at separation 6 m
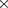
4 m (see Figure 2a). Disease progress was assessed by detailed visual inspection at three monthly intervals for the first five years of the experiment, followed by additional more irregular surveys for two years thereafter. The data consist of the visible disease status of each grapefruit plant in the experimental grove at each survey time; i.e. a series of maps showing which hosts were susceptible and which were (visibly) infected on each survey. However, since surveys were separated by at least three months, and because the 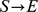 transition is not visible, exact transition times of individual plants are unknown. We therefore fitted the model in Equations 4 and 5 using Markov chain Monte Carlo with data augmentation to estimate the model parameters of interest (i.e. 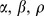 and 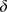) [22], [69], treating the unobserved times as additional nuisance parameters to be estimated. Posterior distributions for the epidemiological parameters could then be obtained *post hoc* by marginalization. Further details of the fitting methodology and expressions for likelihood functions are given in the Text S2.

### Simulating disease progress without control

One thousand independent simulations of the model were performed to assess how BBSC would spread in a typical grove (i.e. 1680 plants at 6 m
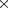
4 m spacing) when disease control is not attempted. We (arbitrarily) took 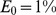, and simulated progression over 20 years, a notional productive lifespan of a citrus grove [63], [64]. Parameter values used in each simulation were drawn randomly from the joint posterior distribution for 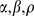 and 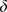 as obtained in estimation. The model was simulated using the Gillespie algorithm [70] (see Text S3 for details).

The number of plants in the central grove that are susceptible at time 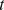 is 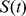, and the number of plants in the exposed compartment is 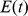. We define the number of asymptomatic plants at time 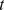 as 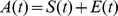. This corresponds to the number of productive (i.e. fruit-bearing) plants at any time. We consider the final number of asymptomatic plants after twenty years, 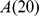, as a simple composite measure of disease spread, corresponding to the productive trees that remain after accounting for the final size of the epidemic over a 20 year period, and we examined the response of this to values of 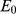 ranging from 0.06% to 2%, i.e. from 1 to 34 initially exposed trees within the central grove. We again used 1000 independent simulations for each initial condition we considered, as we did for each set of parameters in each of the scenarios described below.

### Planting density

To test the effect of host density on disease dynamics, the within-row and between-row spacing of trees were altered, while constraining the total number of trees in the central grove to remain fixed at 1680. The ratio of horizontal to vertical separation was held fixed at 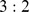 throughout. Again we focused on the final number of asymptomatic plants (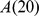) in a grove with 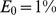, and considered planting densities from 50 to 500 plants per hectare.

While this approach illustrates the effect of inter-host distance on disease spread, it is an oversimplification, since fixing the number of trees at different planting densities corresponds to groves with different areas. To examine the trade-off between disease prevention and productivity we therefore considered the density of asymptomatic trees at 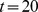 years in the central grove as a function of host density, again for 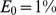.

### Roguing

We modelled a programme of scouting for disease symptoms and roguing detected infected plants. This was included in the model by simulating the examination of every surviving plant in the central grove every 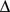 units of time, and independently detecting symptomatic (i.e. class I) plants with probability 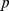. Any detected plants were immediately removed. We considered roguing intervals, 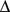, between 7 days and 2 years, and took the probability of detection on a round of scouting to be 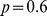, supported by data from Belasque *et al.* [71]. Again we assessed the efficacy of control by examining the value of 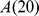, the number of productive trees in the central grove after twenty years.

We considered the responses of 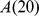 to the roguing interval (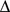) with fixed 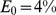, and to 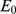 with fixed 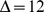 months. We also considered the response of the median value of 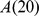 and of the probability of eradicating the pathogen within twenty years as both 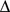 and 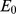 were varied simultaneously. Since the default detection probability 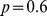 is an estimate, we also considered the sensitivity of our results to this choice, by considering the response of the median value of 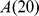 as 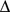 and 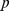 were simultaneously varied.

#### Optimising roguing

As the roguing interval (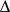) becomes shorter, control improves, and so the yield of the grove increases. However, since scouting then happens more frequently, more plants would need to be examined over the entire lifetime of the grove. The increased number reflects the increased frequency of visits, but also increased numbers of healthy plants associated with improved control. We therefore examined the trade-off between improved yield and additional detection costs by searching for the roguing interval that maximises a measure of the overall profitability of the central grove.

Assuming yearly harvesting from all adult trees aged three years or older at the end of each year [72], the cumulative number of trees that would be harvested (the “(Y)ield”) over our 20 year time scale is given by
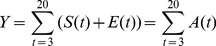
(6)where 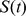 and 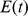 are the numbers of susceptible and exposed plants within the central grove at time 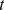, and (as before) the sum 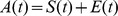 is the total number of productive plants. If the roguing interval is 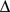 (years), the number of rounds of scouting that occur over the twenty year period is 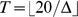. Since removal is immediate and because removed trees do not need to be examined, the total number of plants that are examined (“(V)isited”) is then
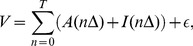
(7)where
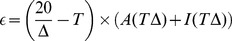
(8)is a correction factor to account for whether or not the roguing interval exactly divides 20 years. If the ratio of the cost of a single examination of a tree for disease symptoms relative to the net profit from the sale price of the fruit from a tree in a single year after cultivation costs have been accounted for is 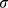, then the profit over the lifetime of the grove will be proportional to 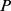, given by



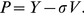
(9)Although a number of factors are omitted from this definition of profitability, including the initial cost of planting the trees, economic discounting, the potential increase in productivity as trees age and so produce more fruit, and the cost of removing diseased trees, we use 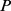 as a simple proxy for the profitability of the central grove.

We first examined the response of 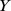 and 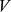 to the roguing interval, 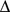, with 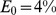. We then examined the profit, 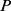, as a function of 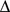, for a range of relative costs of surveying, 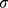. There was an optimal roguing interval, in the sense of a well-defined value of 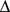 that maximises 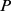, for all values of 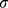. We therefore further examined the response of this maximum profit, and the optimum roguing interval at which it was attained, to the value of 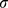, for different levels of initial infection, 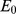.

## Results

### Epidemiological parameters for the experimental data

#### Goodness of fit

Goodness of fit was tested by simulating the model [73] 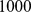 times on a system with the same topology and initial conditions as in the experiment, with model parameters 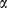, 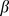, 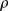, and 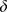 sampled from the estimated joint posterior distribution independently for each simulation. Experimental data for disease progress over time (Figure 2(b)) fell consistently within the range (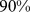 credible interval) of the predicted epidemic trajectories. The temporal evolution of the spatial pattern of disease is summarised (Figure 2(c)) by plotting the proportion of symptomatic trees for each row at each of the discrete survey times. The observed spatial pattern (blue shading) is in good agreement with the pattern from the simulation runs (red). The alternative model without the delay 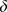 to allow the plants to reach epidemiological maturity was a very poor fit to both the temporal and spatial aspects of the experimental data, and so the inclusion of this extra parameter in our model was judged to be appropriate (data not shown).

#### Estimates of epidemiological parameters

The dispersal scale parameter, 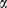, was estimated to have median 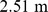, with 95% credible interval 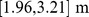. Our estimate of the median average dispersal distance of BBSC is therefore 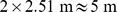. The 95% interval for the rate of infection, 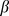, was 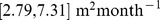, with median 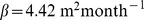. Since the dispersal kernel at distance 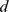 is 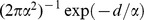 (Equation 4), the average force of infection on a single susceptible plant in the default typical grapefruit grove from a neighbouring single infected plant in the same column is 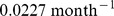. This corresponds to an average time until infection of about 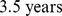. Of course this estimate does not account for the fact that a single infected has more than one neighbour, that there is more than one route of infection apart from nearest neighbour spread, and that there will almost always be more than one infectious plant. Nevertheless, it does indicate that the progression of BBSC in the typical citrus grove we consider is likely to be relatively slow.

The 95% credible interval for the rate of emergence of symptoms, 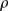, was 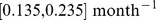, with median 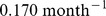. This corresponds to an average incubation period of 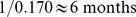. Since symptomatic plants are infectious in our model, this also corresponds to our estimate of the pathogen's latent period. The delay before the pathogen could spread, 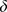, had 95% interval 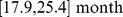, and median 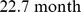.

**Figure 3:**
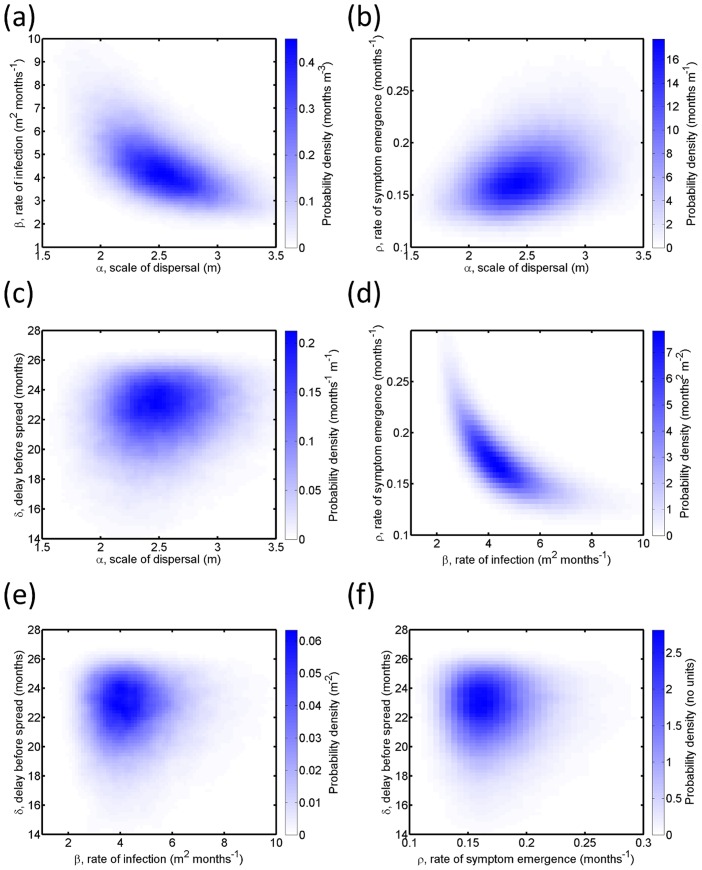
Posterior distributions of parameters. (a)-(f) Pairwise joint posterior distributions for the scale of dispersal, 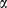; the rate of infection, 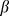; the rate of emergence of infectivity, 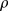; and the delay for plants to reach epidemiological maturity, 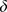. These estimates were obtained by fitting to the experimental data via MCMC with data augmentation. 95% credible intervals: 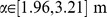, 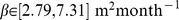, 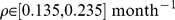 and 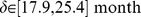.

Pairwise posterior distributions (Figure 3) reveal a strong negative correlation between 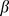 and 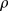. This was expected: if hosts become infectious more quickly, the rate of infection does not need to be so large to lead to the same amount of disease spread. There were also smaller correlations between 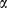 and 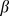 and 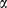 and 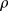. However, since we sample from the joint posterior distribution of all four parameters on each run of the model, we account for any effect of correlations between those pairs of parameters that are associated.

### Disease progress without control

**Figure 4:**
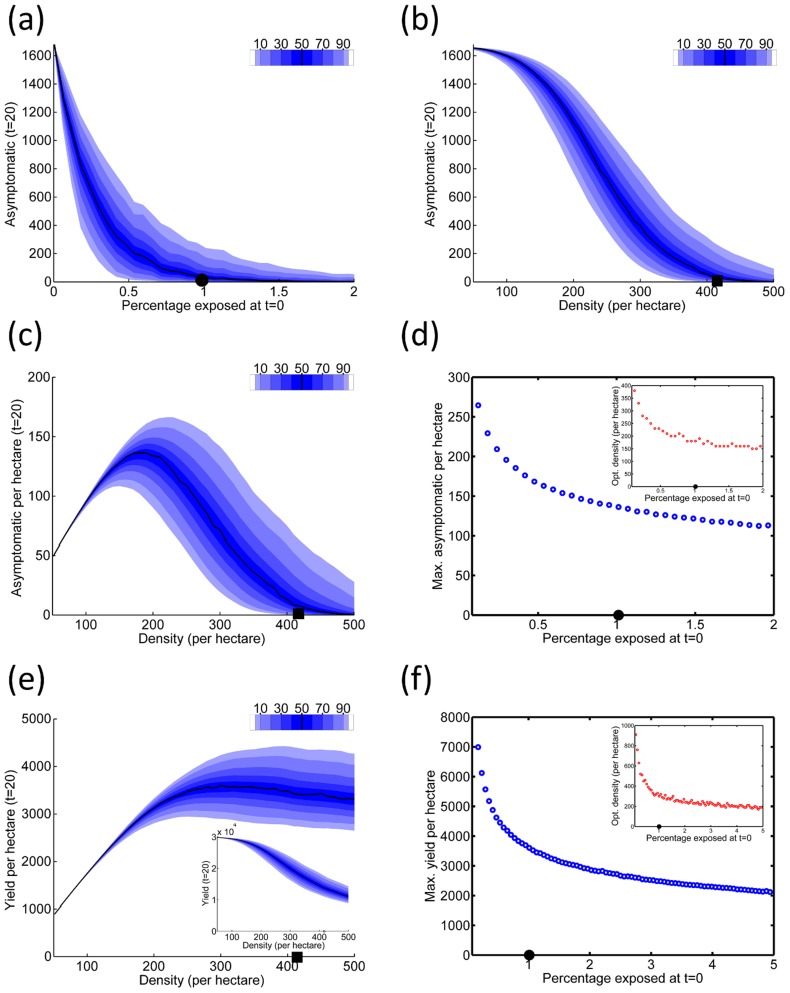
Initial infection and planting density. (a) 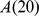, the number of asymptomatic plants (out of a total of 1680 plants) after twenty years, as a function of the percentage of trees that are infected initially, 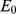. (b) 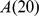 as a function of the density of hosts, when 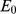 is held fixed at 1%. (c) As Figure 4(b), but showing 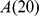 per hectare. (d) The maximum 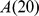 per hectare (the inset shows the planting density at which this optimum is attained) for a range of values of 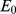. (e) The yield per hectare as a function of the density of hosts, when 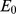 is held fixed at 1%. The inset shows the response of the yield before applying the normalisation by area (i.e. the inset is analogous to (b)). (f) The maximum yield per hectare (the inset shows the planting density at which this optimum value is obtained) for a range of values of 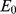. The black symbols on the x-axis of each graph mark default values that are invariant in other scans (i.e. Figures 4(b), 4(c) and 4(e) have 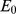 fixed at 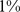; Figure 4(a) shows results for 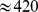 trees per hectare (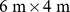)).

Although the disease initially spreads rather slowly, almost all plants within a typical grove are expected to become symptomatic within 20 years when the initial level of infection 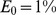 (Figure 1(b)). On average 50% of plants become symptomatic within approximately the first 10 years. Spatial snapshots from an arbitrarily chosen run of the model (Figure 1(c)) indicate that disease spread is very localised, with infection apparently being transmitted largely (but not exclusively) between neighbouring pairs of plants. It also appears to be rather difficult for the pathogen to escape the central grove and to infect plants in the surrounding groves, although this does happen occasionally. Snapshots from other runs indicate that these aspects of BBSC dynamics are general for 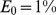; spread is localised with separate foci of infection that grow and coalesce over time, and spread is also largely restricted to the central grove, at least for the first 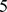 to 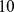 years. Varying the initial level of infection indicates the final number of productive (i.e. asymptomatic) plants at 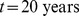, 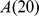, is highly dependent on 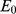 (Figure 4(a)), at least for low values of 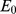. However, since 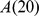 decreases sharply with the amount of inoculum that is initially present, effectively the whole of the central grove becomes infected by 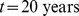 for 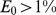.

### Optimising the planting density

The value of 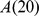 depends strongly on the planting density (Figure 4(b)), with low host density leading to very little spread and so high values of 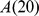 (again with 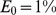). However at more realistic planting densities the spread is much more devastating. On average only 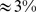 of plants escape (visible) disease by 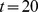 years at the density of the typical grove 
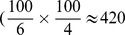
 plants per hectare).

This behaviour leads to a disease-driven trade-off in the number of productive plants per hectare. Low planting density can give excellent disease control, with very high values of 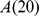, but of course also implies fewer plants per hectare. The optimum density of productive plants is therefore recovered at an intermediate host spacing: for 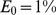, this was at a planting density of around 200 plants per hectare, with 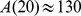 per hectare (Figure 4(c)). This qualitative result is robust to the initial level of infection, and there was an optimum planting density for all values of 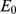 we considered. However both the optimal planting density, and 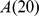 per hectare at this planting density, decreased as the initial level of infection was increased (Figure 4(d)), although these responses begin to flatten off for 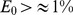.

We also considered the response of the yield (cf. Equation 6) to the planting density. Again for a given level of initial infection, a planting density that leads to an optimum yield per hectare can be defined (Figure 4(e)), although the density that optimises yield when 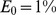 (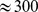 plants per hectare) is larger than that required to maximise the value of 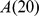 (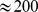 plants per hectare, as described above). The response was also differently shaped, with the yield per hectare remaining at a non-zero value for even very large planting densities (compare 4(c) with 4(e)). This is because even at high densities the epidemic does not infect the entire central grove within the first few years of the epidemic, and so the yield is then non-zero (see also the inset to Figure 4(e), which shows the yield before normalisation of to fixed grove area). However, the response of the optimum planting density required to optimise yield per hectare for different values of the initial level of infection, and the response of the optimum yield per hectare itself at optimum planting density to the initial level of infection both follow a similar pattern to the responses for 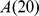 (compare Figure 4(d) and Figure 4(f)).

### Roguing and eradication

**Figure 5:**
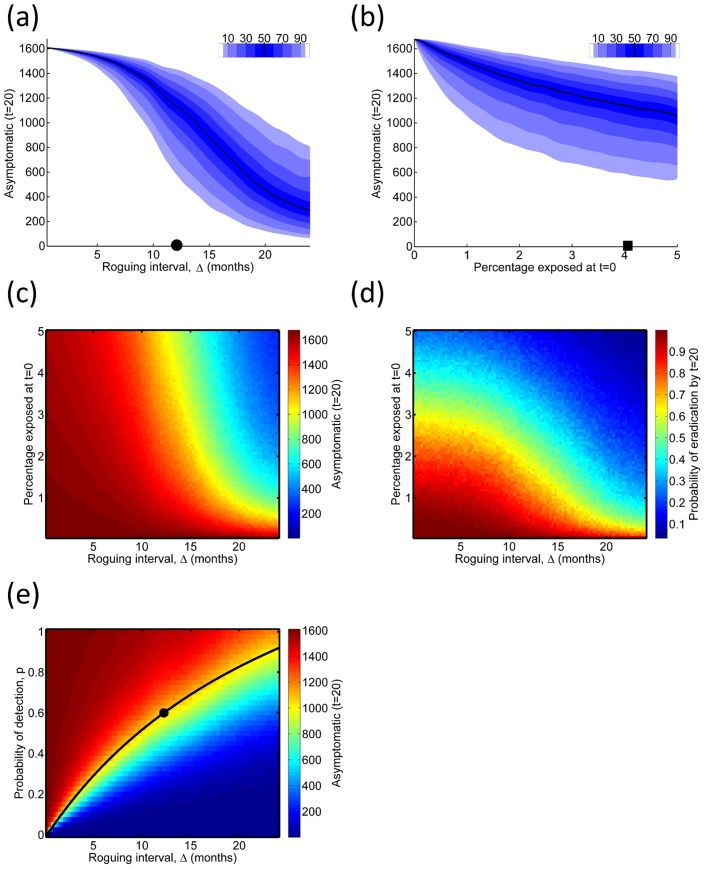
Roguing. (a) 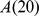 as function of 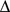, the roguing interval, with initial level of infection 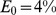. (b) 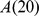 as a function of 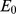, with 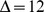 months. (c) The median value of 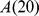 as a function of 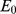 and 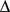. (d) The probability the pathogen is eradicated as a function of 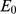 and 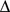. (e) The median value of 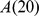 as a function of 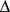 and 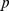, the probability of detecting a symptomatic plant in a single survey, for fixed 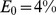. The black curve links pairs of values of 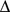 and 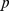 for which the effective infectious period, 
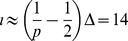
 months (i.e. the value of 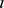 for the default pair of values 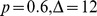 months, which is marked with the black dot).

Even at relatively high initial levels of infection, 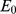, roguing can lead to excellent disease control (Figure 5(a)). At 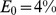 (a level at which every plant within the central grove would become infected without control within 20 years), even the rather long roguing interval 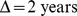 would save approximately 20% of plants from visible symptoms at 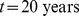. As 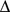 is shortened, 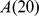 of course increases. Values of 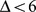 months lead to high levels of disease control (e.g. 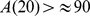
%), and even 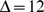 gives 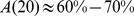. This response is comparatively robust to the initial level of infection (Figure 5(b)): although 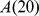 does decrease as 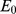 is increased (for fixed 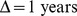), it does so only relatively slowly.

The value of 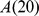 in fact always depends on 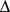 and 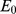 in this broad fashion (Figure 5(c)), decreasing as either parameter is increased. For short roguing intervals, however, 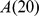 was relatively irresponsive to 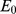, and indeed there was a large set of 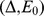 pairs for which excellent control was achieved. This was despite the more restricted range of pairs of these parameters for which the pathogen was reliably eradicated from both the central and the surrounding groves (Figure 5(d)).

We also examined the response of the median value of 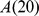 to changes in the roguing interval, 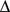, and the probabilty of detection, 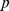 (Figure 5(e)). Unsurprisingly, the impact of the epidemic is increased as 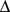 is increased or 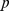 is decreased. In fact the shape of the contours of constant 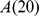 can be explained by a simple calculation. If the other epidemiological parameters are fixed, the efficacy of roguing is set by the effective infectious period of the average host. This is the time for which the host is infectious, i.e. the time between the emergence of infectivity after the latent period has passed and later removal of the host by roguing. If the probability of detection is 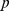, then the number of surveys required to detect a host after the emergence of symptoms upon it is a geometric random variable, with average 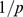. A particular symptomatic plant could have become infectious at any time between the final round of surveying when it was asymptomatic/uninfectious and subsequent round by which time it was symptomatic. If we assume the time of the transition between states 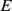 and 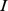 in our model is uniformly distributed between surveys (i.e. if we ignore any knock on effect from the slight increase in the rate of infection between rounds of detection that would occur because the number of infected plants increases between surveys), then the average effective infectious period can be approximated by
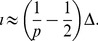
(10)


For the default parameters 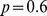 and 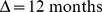, the average infectious period is 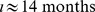; all 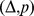 pairs with this effective infectious period are shown by the black curve in Figure 5(e).

#### Optimising roguing

**Figure 6:**
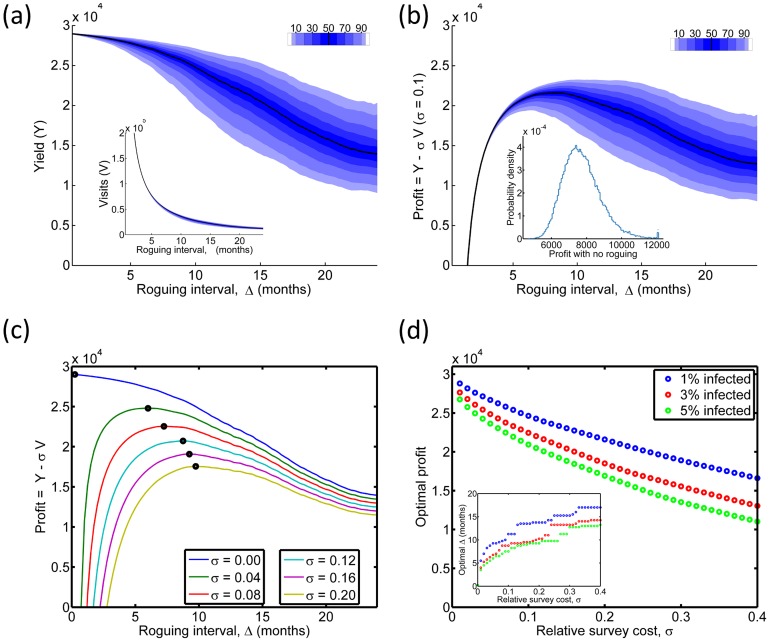
Economics of roguing. (a) Responses of the total yield, 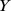, and the total number of visits to individual plants, 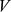, (shown in inset) both over the twenty year nominal lifetime of the grove, to the roguing interval, 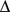. The initial level of infection 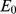 was 4%. (b) The profit, 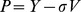, as a function of 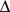, when the relative cost of surveying, 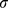, is fixed at 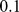, again for 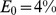. The distribution of profit when there is no roguing is shown in the histogram in the inset. (c) Responses of 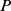 to 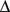 for different values of 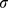. The black dots denote the roguing interval for which maximum profitability was attained. In all cases 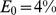. (d) The maximum 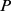, and the value of 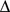 at which this maximum profit was attained (inset), for a range of values of 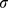 and for different values of 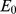.

Both the yield, 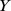, and the cost of surveying, 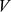, decrease as the roguing interval, 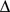, increases (Figure 6(a)). However, as 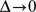, the cost of surveying increases without bound, meaning that for relative cost of surveying 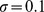, the profit (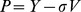) has a well defined maximum at 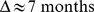 (Figure 6(b)). The qualitative result – i.e. that there is a roguing interval at which profitability is maximised – holds for all values of 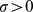 we considered (Figure 6(c)). Unsurprisingly, as 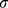 increases, the optimal value of 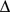 increases, and 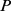 decreases, irrespective of 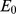 (Figure 6(d)).

## Discussion

We used Markov chain Monte Carlo with data augmentation to fit a spatially-explicit, stochastic, epidemiological model to the spread of BBSC, and have estimated a number of key epidemiological parameters. Dispersal was exponential, with median approximately 5 m (similar to the distance between neighbouring pairs of plants in a typical citrus grove in Brazil). Laranjeira *et al.* [20] suggest that the BBSC pathogen may be transmitted by an air-borne vector of limited dispersion ability, and our results are consistent with that possibility. Our estimate of the dispersal scale, together with a careful review of the dispersion ability of arthropods detected in the Bahia region, may help to narrow the set of candidate vectors. Certainly a number of mites and scale insects are known to transmit viral diseases, both in citrus [74] and other perennials [75], and similar species would be an obvious place to begin such a search. Our parameter estimates are also consistent with an association between a bark wounding insect and a splash dispersed fungus.

To obtain an adequate fit to the experimental data we included a delay for plants to reach epidemiological maturity before being able to spread and/or show symptoms of the disease in our model. While it is of course rather difficult to give a mechanistic interpretation of this delay because of the uncertainities surrounding BBSC etiology, it could, for example, correspond to a need for mature tissues for symptom expression, or a bark borer insect vector that only feeds on mature bark. Irrespective of its mechanistic basis, our estimate of the delay is approximately 24 months. Laranjeira *et al.* [20] took the long delay before disease began to spread in the experiment as indicative of the incubation period for the pathogen that causes BBSC, which we instead estimated to be approximately 6 months. Given the very good statistical support for our model fitting, we contend that our new interpretation of the experimental results is more plausible, especially since a two year incubation period is rather long for a vectored disease.

In a grove at planting density typical of citrus production in Brazil, we predict that BBSC would spread slowly. This was unsurprising given the relatively slow rate of disease spread in the original experiment, in which the density of host plants was approximately six times higher than found in citrus production. Nevertheless, and slow spread notwithstanding, we predict BBSC would easily spread throughout an entire grove within 20 years, even for modest levels of initial infection (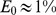). In turn this indicates that careful sanitation of new plantings for BBSC symptoms is important. Despite the official programs to foster propagative plants under screenhouses in Bahia, symptomatic “mother” plants are still found [62], and most nurseries are not kept under screenhouses [76]. This clearly presents a risk, particularly since there is no diagnostic test to identify asymptomatic infected plants. This compelled us to investigate other types of control apart from sanitation. We note that, although high BBSC severity and incidence can be routinely detected in mature commercial groves in Bahia, the incidence of disease is usually quite low at the time of first detection (HP Santos-Filho, personal communication). The particular range 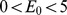 we used was therefore intended to account for the full range of values that may occur in practice, given groves at different distances from sources of inoculum and/or with different levels of sanitation before planting. The influence of the initial level of infection on the optima we identify indicates that, for practical implementation, it would be advantageous to perform further experimentation and/or further data-collection to enable 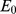 to be more precisely quantified.

We therefore used our model to examine the effect of host spacing on disease spread. As the density of hosts was increased, so did the level of disease, which of course was expected [26]. However this is particularly unfortunate given recent trends toward higher planting densities in commercial citrus production in Brazil [77]. We therefore examined the trade-off between host density and productivity in the presence of disease by considering the density of plants that escape infection over a 20 year timescale as the host spacing was altered. We found an optimum planting density, at which the reduction in productivity due to planting fewer hosts per hectare was offset by the reduced losses to disease (cf. Figure 3(c)). Although the exact nature of this optimum depended on the initial level of infection, optimal densities were typically sufficiently low that there would be enough space for an intercrop to be established. This approach is already used in Brazil, where growers sometimes plant passion fruit or pineapple between rows of citrus. However, since the intercrop would undoubtedly have its own effect(s) on pathogen dispersal [32], [33], investigating the epidemiological consequences of intercropping requires more data.

According to our simulation results, roguing, even when detection is imperfect, can control disease successfully (cf. Figure 4). Control can be achieved for relatively long roguing intervals, even for high levels of initial infection. Indeed in our scans showing the effect of roguing interval on control efficacy we used a default value of 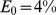 (rather than 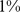 as used in assessing the effect of host density) in order to obtain a more meaningful response as the parameters of interest were changed. This good level of control was possible because of the slow rate of BBSC spread and its limited dispersal ability. Control by roguing is also aided by the absence of cryptic infection (i.e. hosts that are able to infect without showing symptoms). This contrasts with a number of other pathogens of citrus, for example *Xanthomonas axonopodis*, the bacterium that causes citrus canker, for which there is both significant long-range dispersal [78] and cryptic infection [6]. Indeed the recent attempt to eradicate citrus canker from Florida involved removing any host plant within 579.1 m (1900 ft) of a detected symptomatic focal plant, irrespective of apparent disease status [79]. However, the epidemiology of BBSC indicates that a similar approach is not required here, and initial tests of this type of control strategy indicated that it did not noticably outperform simple roguing (data not shown).

Control was possible even though roguing only occurred within the central grove. It did not require the pathogen to be entirely eradicated from the system, and indeed for high values of 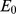, the pathogen was eradicated only rarely (cf. Figure 5(d)), presumably because there was at least one escape of the pathogen from the central grove before it was effectively controlled there. This surprisingly high level of control despite an ever-increasing external reservoir reflects the low probability of the pathogen returning to the central grove once it has escaped (cf. Figure 1(c)), and on the occasions it does return, frequent roguing limits its impact. Ultimatately this derives again from the limited disperal ability of the pathogen that causes BBSC. For pathogens capable of faster and/or long-distance dispersal, synchronisation in control is acknowledged to be extremely important, since otherwise the pathogen is able to persist, bulk-up and repeatedly cause devastating reinvasion from uncontrolled areas that act as refugia [10]. Following common practice in the Brazilian citrus industry, removed plants were not replaced in our model, which again facilitated control. Replanting removed trees results in a constant supply of new susceptible hosts to areas with infection, which necessarily makes control more difficult.

The efficacy of roguing was characterised by considering 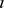, the average effective infectious period (Equation 10), and this quantity was an excellent predictor of the number of plants that escape disease (cf. Figure 5(e)). Investigating how this result generalises to pathogens that are harder to control would be an interesting extension, particularly because the approximation used in the calculation of 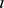 is most accurate for pathogens that spread slowly. We note that, although simple, the principle underlying the calculation of 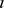 has been reported incorrectly in previous studies that used non-spatial, compartmental models. Parameter values given in Table 2 of Jeger *et al.* [3] (see also Madden *et al.* [80]) indicate that if roguing is performed monthly then the equivalent removal rate would be 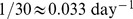. This assumes that symptoms and infectivity are developed immediately after rounds of surveys, and so that the average infectious period is 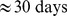. Given the more accurate estimate of 15 days, the rate of removal for monthly surveys with perfect detection should in fact be 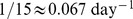.

By introducing a simple measure of the profitability of a grove, we demonstrated the trade-off between the cost of detection and the benefits of control (cf. Figure 6). An optimum roguing frequency can be determined, balancing the increased cost of roguing more frequently against the improved control it leads to, although this optimum is conditioned on the initial level of disease (cf. Figure 6(d)) and the cost of examining a plant for disease symptoms relative to the difference between the sale price of the fruit from a single year's harvest and the yearly cost of cultivating a tree.

For simplicity and ease of presentation, our definition of the cost of control focused exclusively on the cost of detection and did not include the cost of removal. However, because an individual plant would potentially be surveyed many times, but can be removed at most once, we believe this is a reasonable simplification. While our methodology could readily be extended to include more complex economics (e.g. removal costs, cost of initial grove establishment, increased yield from older plants), or to allow for growers potentially ceasing cultivation if the net profit from a particular grove fell below zero despite the yield that would subsequently accrue, the broad result would certainly be robust to these changes. A more interesting extension would be control strategies that change over time. An example of this is a roguing interval that depends on the current (observed) prevalence of infection, and so that could cause surveying to slow down or even stop once the disease was judged to be under control. This differs from the implementation considered here, in which the cost of detection for low levels of initial infection and short roguing intervals may be overstated: any grower who surveyed weekly but did not find disease for a number of years would doubtless reduce the frequency of surveying or even stop entirely. Investigating this type of adaptive strategy, together with the consequential risk of failure that derives from having to predict whether the disease has actually been eradicated or has merely not been found recently, will form the basis of our future work in this area.

A number of previous models have used deterministic mean field representations of cultural control [1], [2], [54], [55], [81]. More recently stochastic, spatially-explicit models have predominated [10], [56]–[58], although typically these models are not fitted to data (a series of studies of the failed eradication of citrus canker in Florida are the exception [6]–[8]). What previous models lack, however, is a treatment of the economic aspects of control, and the trade-offs and optima to which this can lead. While significant progress in examining this type of trade-off has been made using optimal control theory [59], [82], [83], the complexity of the associated mathematics has necessarily reverted attention to deterministic, non-spatial models. Using a spatial, stochastic model parameterised with real data to balance the benefits of effective disease control against its costs is the novel aspect of our work. In addition to the additional insight into BBSC epidemiology obtained by our model fitting, providing a “real world” example showing how a mathematical model can be used to optimise and test both the epidemiological and economic aspects of control strategies for a plant disease is therefore the key contribution of this paper.

## Supporting Information

Table S1 **Symbols, definitions and typical values for variables and parameters**.
(PDF)

Text S1 **Experimental data from Laranjeira** ***et al.*** **(2006)**.
(XLS)

Text S2 **Parameter estimation**.
(PDF)

Text S3 **Simulation algorithm**.
(PDF)
