# Supplementary material for: Cost-Effective Control of Plant Disease When Epidemiological Knowledge Is Incomplete: Modelling Bahia Bark Scaling of Citrus
Source: PLoS Comput Biol. 2014 Aug 7;10(8):e1003753. doi: 10.1371/journal.pcbi.1003753 (PMC4125052; doi:10.1371/journal.pcbi.1003753)
Supplement: Table S1 — Symbols, definitions and typical values for variables and parameters. (PDF) [file pcbi.1003753.s001.pdf]

Table S1. Symbols, definitions and typical values for variables and parameters

Table 1. The ranges of values for α, β, γ and δ are 95% credible intervals as obtained in estimation. The effective infectious period, ι, is the average time for which a host is infectious if roguing occurs. The relative cost of surveying, σ, is the ratio of prices of examining a single plant once for symptoms of disease to the difference between the sale price of the fruit harvested from a healthy tree and the cost of its cultivation over a single year. The correction factor ϵ accounts for roguing intervals Δ that are not exactly divisible into the 20 year period we examine (cf. Equation 8 in the main text).

Symbol	Description	Value/Definition
t	Time since initial planting	-
$S(t)$	Number of susceptible plants	-
$E(t)$	Number of exposed plants	-
$I(t)$	Number of infected plants	-
$R(t)$	Number of removed plants	-
$A(t)$	Number of asymptomatic plants	$A(t) = S(t) + E(t)$
E_0	Percentage of plants exposed at $t = 0$	varied (default 1% or 4%)
ϕ_i	Rate at which i^{th} host becomes infected	$\beta \sum_{j \in \Omega_I} K(d_{ji}; \alpha)$
Ω_S	Set of susceptible hosts	-
Ω_E	Set of exposed hosts	-
Ω_I	Set of infectious hosts	-
$K(d_{ij}; \alpha)$	Dispersal kernel	$(2\pi\alpha^2)^{-1} \exp(-d/\alpha)$
d_{ij}	Distance between hosts i and j	-
α	Dispersal scale (mean 2α)	$[1.96, 3.21]$ m
β	Rate of infection	$[2.79, 7.31]$ m^2 month^{-1}
ρ	Rate of onset of infectiousness/symptoms	$[0.135, 0.235]$ month^{-1}
δ	Delay before epidemiological maturity	$[17.9, 25.4]$ month
Δ	Roguing interval	varied (default 1 year)
p	Probability of detection	varied (default 0.6)
ι	Effective infectious period	$\iota \approx \left(\frac{1}{p} - \frac{1}{2}\right)\Delta$
σ	Relative cost of surveying	varied (default 0.1)
T	Total number of surveys	$T = \lfloor 20/\Delta \rfloor$
V	Total number of trees surveyed	$V = \sum_{n=0}^{T} (A(n\Delta) + I(n\Delta)) + \epsilon$
ϵ	Correction factor	$\epsilon = \left(\frac{20}{\Delta} - T\right) \times (A(T\Delta) + I(T\Delta))$
Y	Total number of trees harvested	$Y = \sum_{t=3}^{20} A(t)$
P	Profit (up to a scale factor)	$P = Y - \sigma V$
