# Supplementary material for: Cost-Effective Control of Plant Disease When Epidemiological Knowledge Is Incomplete: Modelling Bahia Bark Scaling of Citrus
Source: PLoS Comput Biol. 2014 Aug 7;10(8):e1003753. doi: 10.1371/journal.pcbi.1003753 (PMC4125052; doi:10.1371/journal.pcbi.1003753)
Supplement: Text S2 — Parameter estimation. (PDF) [file pcbi.1003753.s003.pdf]

Text S2. Parameter estimation

The model parameters were estimated using Bayesian inference [1]. The posterior distribution for the parameters is given by Bayes' formula

$$\pi\left(\alpha,\,\beta,\,\rho,\,\delta|D\right)\propto P\left(D|\alpha,\,\beta,\,\rho,\,\delta\right)\pi\left(\alpha,\,\beta,\,\rho,\,\delta\right),$$

where D represents the data, $\pi\left(\alpha,\,\beta,\,\rho,\,\delta\right)$ is the joint prior distribution for the parameters (reflecting our knowledge prior to experimental observation), and $P\left(D|\alpha,\,\beta,\,\rho,\,\delta\right)$ is the likelihood (the probability of the data given the parameters).

The likelihood can be written down explicitly in the case of uncensored data, D^{unc}, when the times of all epidemiological transitions are known (i.e. the time of infection t_i^E and the time to symptoms t_i^I for each host, i). The "uncensored" likelihood, $P(D^{\mathrm{unc}}|\alpha,\,\beta,\,\rho,\,\delta)$, is the product of three types of contributions; from trees that at the time of the final survey ($t_{\mathrm{end}}=82$ months) are (i) infectious, denoted by $\mathrm{I}(t_{\mathrm{end}})$; (ii) infected but not infectious, $\mathrm{E}(t_{\mathrm{end}})$; and (iii) still susceptible, $\mathrm{S}(t_{\mathrm{end}})$

$$\begin{aligned}P\left(D^{\mathrm{unc}}|\alpha,\,\beta,\,\rho,\,\delta\right)=&\prod_{i\in\mathrm{I}(t_{\mathrm{end}})}\phi_i(t_i^E)\exp\left(-\int_\delta^{t_i^E}\phi_i(u)du\right)\rho\exp\left(-\rho\left(t_i^I-t_i^E\right)\right)\\&\times\prod_{j\in\mathrm{E}(t_{\mathrm{end}})}\phi_j(t_j^E)\exp\left(-\int_\delta^{t_j^E}\phi_j(u)du\right)\exp\left(-\rho\left(t_{\mathrm{end}}-t_j^E\right)\right)\\&\times\prod_{k\in\mathrm{S}(t_{\mathrm{end}})}\exp\left(-\int_\delta^{t_{\mathrm{end}}}\phi_k(u)du\right).\end{aligned}\tag{1}$$

Here, $\phi_i(t)=\beta\sum_{j\in\mathrm{I}(t)}K\left(d_{ij},\,\alpha\right)$ is the hazard function for tree i at time t (cf. Equation 5 in the main text).

In our case, however, the exact transition times are unknown, and the likelihood is

obtained from (1) by integrating out the unobserved times

$$P\left(D|\alpha,\,\beta,\,\rho,\,\delta\right)=\int_{\mathbf{T}}P\left(D^{\mathrm{unc}}|\alpha,\,\beta,\,\rho,\,\delta\right)\mathbf{dt^{E}dt^{I}},$$

where the integral is carried out on the space $\mathbf{T}$ spanned by the unobserved times consistent with the observational data. This integral is not analytically tractable, and was instead calculated numerically within an MCMC routine using data augmentation. The unobserved times $\{t_i^E\}$, $\{t_i^I\}$ were therefore treated as nuisance parameters to be estimated in parallel with the model parameters of interest (i.e. α, β, ρ, δ) [2,3].

The joint posterior distribution for the augmented set of parameters was estimated using the Metropolis-Hastings algorithm [1]. Independent prior distributions were used for all parameters: uniform priors were used for α (with support $[0, 10^3]$ m), β (with support $[0, 10^3]$ m^2 month^{-1}), and δ (with support $[0, 26]$ month, corresponding to the period for which no symptomatic hosts were observed). For the inverse incubation period, ρ, we used a weakly-informative exponential prior with mean $1/12$ month^{-1}, informed by previous estimates [4]. Prior distributions for all augmented times of individual transitions were uniform over the support consistent with the observational data.

Each Monte Carlo chain was run for 5×10^5 iterations and was monitored for convergence, with an initial burn-in period of 5×10^4 iterations discarded from the analysis. Different initial values of the parameters were used, in order to check that the final distribution was not sensitive to the initial conditions.

References

1. Gilks W, Richardson S, Spiegelhalter D (1995) Markov Chain Monte Carlo in Practice. Chapman and Hall.

2. Gibson G, Renshaw E (1998) Estimating parameters in stochastic compartmental models using Markov chain methods. IMA Journal of Mathematics Applied in Medicine and Biology 15: 19–40.

3. O'Neill P, Roberts G (1999) Bayesian inference for partially observed stochastic epidemics. Journal of the Royal Statistical Society A 162: 121–129.

4. Laranjeira F, Barbosa C, Santos Filho H, Gonçalves T, Nickel O (2006) Progress, spread and natural transmission of Bahia bark scaling of citrus in Brazil. Annals of Applied Biology 148: 187–195.
