# Supplementary material for: Cost-Effective Control of Plant Disease When Epidemiological Knowledge Is Incomplete: Modelling Bahia Bark Scaling of Citrus
Source: PLoS Comput Biol. 2014 Aug 7;10(8):e1003753. doi: 10.1371/journal.pcbi.1003753 (PMC4125052; doi:10.1371/journal.pcbi.1003753)
Supplement: Text S3 — Simulation algorithm. (PDF) [file pcbi.1003753.s004.pdf]

Text S3. Simulation algorithm

Simulations must account for both types of event that can affect the disease status of individual plants.

1. "Epidemiological". Transitions due to the spread of disease, i.e. infection (affects susceptible plants) or emergence of infectivity/symptoms (affects exposed plants). These transitions occur at rates depending on the current state of the system.

2. "Human-mediated". Transitions due to control intervention, i.e. removal (of symptomatic plants). These transitions (potentially) occur every Δ units of time, where Δ is the roguing interval.

Timing of epidemiological transitions was determined using the (direct) Gillespie algorithm [1]. A single epidemiological event occurs at overall rate given by the sum of the rates of the individual events, i.e. i) infection, which occurs at rate ϕ_i for each uninfected host i, and ii) emergence of infectivity, which occurs at rate ρ for each exposed host. If Ω_S is the set of susceptible hosts, and Ω_E the set of exposed hosts, the total rate of any epidemiological transition is therefore $\Upsilon = \sum_{i \in \Omega_S} \phi_i + |\Omega_E|\rho$, where $|\Omega_E|$ is the number of exposed hosts. A sample from an exponential distribution with rate parameter Υ leads to the delay, t^*, after which the next such event would occur if the system were to remain unperturbed.

If an epidemiological event is scheduled to occur before any potential removal of hosts due to control (which happens on a regular schedule every Δ units of time), the epidemiological event actually occurs after a delay of t^* units of time, and the identity of the host that transitions is determined randomly in proportion to the rates of the individual events that constitute Υ. If, however, a potential removal and so change in rates due to a perturbation is scheduled for time t^e, with $t^e < t + t^*$, where t is the current time, then

the host is removed and the rates simply updated. A new value of Υ and in turn t^* is then determined, after updating the current time to be $t = t^e$. This procedure generates a statistically correct trajectory since all epidemiological events follow exponential distributions in our model [2], and so waiting times are independent of any time that has already passed.

References

1. Gillespie D (1977) Exact stochastic simulation of coupled chemical reactions. Journal of Physical Chemistry 81: 2340–2361.

2. Cunniffe N, Stutt R, van den Bosch F, Gilligan C (2012) Time-dependent infectivity and flexible latent and infectious periods in compartmental models of plant disease. Phytopathology 102: 365-380.
